# Prediabetic amylin hypersecretion impairs brain glucose regulation

**DOI:** 10.1002/alz70855_105720

**Published:** 2025-12-24

**Authors:** Ravichandra S Davargaon, Deepak Kotiya, Noah S Leibold, Nirmal Verma, Huazhen Liu, Pradeep Kachroo, Florin Despa

**Affiliations:** ^1^ University of Kentucky, Lexington, KY, USA

## Abstract

**Background:**

Type‐2 diabetes‐related hyperglycemia increases the risk for cognitive decline. Amylin is a centrally‐linked pancreatic hormone that induces satiation. The beneficial metabolic effects of amylin led to regulatory approval of the amylin analog drug, pramlintide, for weight loss. Different research teams (including ours) report amylin co‐aggregates with brain parenchymal and vascular β‐amyloid in persons with Alzheimer's dementia. Using cancer cells, other teams reported that amylin inhibits glycolysis. The present study sought to determine changes of glucose utilization in brain tissues associated with suppressed vs. oversecreted pancreatic amylin.

**Method:**

Because murine amylin is not amyloidogenic, we generated mice “humanized” for amylin expression (hA^ON^ mice). Amylin knock‐out mice (hA^OFF^ mice) and mice expressing wild‐type mouse amylin (WT mice) served as controls. All mouse groups underwent four‐month overnutrition to induce prediabetic amylin hypersecretion (in hA^ON^ and WT mice), followed by endpoint novel object recognition and mass spectrometry analyses of brain tissue glucose 6‐phosphate (G6P) (the first intermediate of intracellular glucose metabolism) and glycolytic amino acids serine, glycine and alanine. We then assessed the ratios of serine, glycine and alanine to G6P (i.e. the glycolytic amino‐acid flux).

**Result:**

Compared to WT and hA^OFF^ males, hA^ON^ males had longitudinal increases of the blood glucose levels (one‐way ANOVA, *p* < 0.0001) and decreased recognition indices (one‐way ANOVA, *p* < 0.0001), at the endpoint. Compared to hA^OFF^ and WT males, hA^ON^ males had higher brain tissue G6P levels and much lower brain tissue glycolytic amino‐acid flux, whereas hA^OFF^ males had the lowest average brain tissue G6P level (one‐way ANOVA, *p* < 0.0001) and highest average brain tissue glycolytic amino‐acid flux (one‐way ANOVA, *p* < 0.0001). Age‐matched males from similar amylin genotype groups that were on a chow diet showed unaltered glucose homeostasis. Compared to mice in chow diet groups, metabolically stressed hA^ON^ and WT males had increased brain tissue amylin levels. Brain amylin accumulation was higher in hA^ON^ vs. WT mice (one‐way ANOVA, *p* < 0.0001) consistent with amyloidogenicity of human amylin.

**Conclusion:**

Prediabetic amylin hypersecretion increases brain amylin level and exacerbates amylin receptor signaling controlling glycolysis, leading to impairments of glycolytic flux and memory.

